# Discovering the direct relations between nutrients and epigenetic ageing

**DOI:** 10.1016/j.jnha.2024.100324

**Published:** 2024-07-26

**Authors:** Pol Grootswagers, Daimy Bach, Ynte Biemans, Pariya Behrouzi, Steve Horvath, Charlotte S. Kramer, Simin Liu, JoAnn E. Manson, Aladdin H. Shadyab, James D. Stewart, Eric Whitsel, Bo Yang, Lisette de Groot

**Affiliations:** a Division of Human Nutrition and Health, Wageningen University and Research, Wageningen, Netherlands; b Biometris, Mathematical and Statistical Methods, Wageningen University and Research, Wageningen, Netherlands; c Department of Human Genetics, David Geffen School of Medicine, University of California, Los Angeles, USA; d Altos Labs, San Diego Institute of Science, San Diego, CA, USA; e Department of Biostatistics, Fielding School of Public Health, University of California, Los Angeles, USA; f Department of Epidemiology and Center for Global Cardiometabolic Health, School of Public Health, Departments of Medicine and Surgery, Alpert School of Medicine, Brown University, Providence, RI, USA; g Department of Medicine, Brigham and Women's Hospital, Harvard Medical School, Boston, MA, USA; h Herbert Wertheim School of Public Health and Human Longevity Science, University of California, San Diego, La Jolla, CA, USA; i Department of Epidemiology, Gillings School of Global Public Health, University of North Carolina at Chapel Hill, Chapel Hill, NC, USA; j Department of Medicine, School of Medicine, University of North Carolina, Chapel Hill, NC, USA; k Department of Epidemiology and Center for Global Cardiometabolic Health, School of Public Health, Brown University, Providence, RI, USA

**Keywords:** Epigenetic ageing, Nutrition, Copula graphical models, Epigenetic clock

## Abstract

**Background:**

Along with the ageing of society, the absolute prevalence of age-related diseases is expected to rise, leading to a substantial burden on healthcare systems and society. Thus, there is an urgent need to promote healthy ageing. As opposed to chronological age, biological age was introduced to accurately represent the ageing process, as it considers physiological deterioration that is linked to morbidity and mortality risk. Furthermore, biological age responds to various factors, including nutritional factors, which have the potential to mitigate the risk of age-related diseases. As a result, a promising biomarker of biological age known as the *epigenetic clock* has emerged as a suitable measure to investigate the direct relations between nutritional factors and ageing, thereby identifying potential intervention targets to improve healthy ageing.

**Methods:**

In this study, we analysed data from 3,969 postmenopausal women from the Women's Health Initiative to identify nutrients that are associated with the rate of ageing by using an accurate measure of biological age called the PhenoAge epigenetic clock. We used Copula Graphical Models, a data-driven exploratory analysis tool, to identify direct relationships between nutrient intake and age-acceleration, while correcting for every variable in the dataset.

**Results:**

We revealed that increased dietary intakes of coumestrol, beta-carotene and arachidic acid were associated with decelerated epigenetic ageing. In contrast, increased intakes of added sugar, gondoic acid, behenic acid, arachidonic acid, vitamin A and ash were associated with accelerated epigenetic ageing in postmenopausal women.

**Conclusion:**

Our study discovered direct relations between nutrients and epigenetic ageing, revealing promising areas for follow-up studies to determine the magnitude and causality of our estimated diet-epigenetic relationships.

## 1 Introduction

While prolonged life expectancy is one of the most noteworthy achievements of humanity, morbidity rates are expected to rise further as ageing is a predominant risk factor for common diseases, such as cancer and cardiovascular disease (CVD) [1,2]. Consequently, the rapidly ageing population faces major healthcare, economic and social implications, which emphasises the importance of healthy ageing [[3], [4], [5], [6], [7]]. However, not everyone ages at the same rate with regard to their health status and susceptibility to disease and death [3,7,8]. Therefore, as opposed to *chronological age*, which merely refers to the time elapsed, *biological age* was coined, considering the degree of physiological deterioration over time [1,7]. The rate of biological age varies among individuals and can be influenced by various factors, including genetics, environmental exposures and lifestyle habits [3,5,8]. As a result, biological age represents ageing more accurately, which is illustrated by its better predictive capacity for morbidity and mortality [1,3,5]. As biological age responds to lifestyle and environmental factors [8], it is a suitable indicator to identify malleable factors that relate to ageing [3,5,9,10].

Emerging research indicates a complex interplay between lifestyle and genetics in influencing ageing, with genetics accounting for a variable but noteworthy 1%–27% of lifespan variation in the population [11]. Alongside recent genome-wide analyses that have identified measurable yet not overwhelming genetic effects on the ageing rate [12,13], lifestyle factors have been proposed to play a more significant role in determining the pace of ageing [5,14]. In particular, various nutritional factors, such as dietary fibre and omega-3 fatty acids along with a physically active lifestyle [10,15,16], have been reported to mitigate age-related disease risk and, therefore, may have the potential to improve healthy ageing [14,17,18]. Yet, despite the strong evidence connecting nutrition to health outcomes, it remains unclear which specific nutritional factors accelerate or decelerate biological ageing [10,17,19].

To determine factors associated with of accelerated or decelerated ageing rates, researchers have been studying clinical biomarkers that can accurately reflect biological age [5,19]. To conceptualise the underlying mechanism of biological ageing, twelve cellular and molecular hallmarks of ageing (e.g., telomere attrition) were identified [5,20]. Among these hallmarks, research suggests DNA methylation (DNAm) as the most accurate biomarker of ageing [5,9,21,22]. DNAm is a process in which dynamic DNA modifications regulate gene expression without changing the DNA sequence [5,21,22]. The most common type of DNAm involves the biochemical process of adding a methyl group to a cytosine nucleotide leading to the formation of a 5-methylcytosine (5mC) [5,9,21]. This methylation is almost exclusively found on a cytosine nucleotide, followed by a guanine nucleotide, together referred to as a CpG-site [5,21]. Abundant studies have demonstrated a close relationship between changes in DNAm on specific CpG-sites and the ageing process [19,23,24]. These age-dependent changes in DNAm include global hypomethylation and region-specific hypermethylation [23,25]. Accordingly, measures named *epigenetic clock*s can estimate biological age based on the cumulative assessment by machine learning algorithms of DNAm at age-related CpG-sites [5,9,21,22,25]. Among these clocks, the PhenoAge clock stands out as the currently most accurate measure of biological age and a strong predictor of both mortality and morbidity [25].

With epigenetic clocks, epigenetic age acceleration can be calculated, which is defined as the relative discrepancy between the biological age measure and chronological age [5,18,19,26]. Overall, research identified that epigenetic age acceleration (i.e. higher biological age than chronological age) is associated with multiple age-related conditions, such as obesity and dementia, whereas epigenetic age deceleration is associated with lifestyle factors, such as a healthy diet and physical activity [3,[26], [27], [28]]. Evidently, epigenetic age acceleration provides valuable insights into the rate of biological ageing and can be used to identify specific nutrients that accelerate or decelerate the ageing process [10,16,28]. Given the novelty of integrating ageing research with nutrition and lifestyle data, adopting an objective exploratory approach is recommended. Instead of focusing on single nutrients in a hypothesis-driven way, the wide spectrum of nutrients calls for a data-driven way to reveal the interactive and complex network, allowing for more open-minded observations [10,25,29,30]. A promising statistical tool for this approach is the Copula Graphical Models (CGM), which is a network analysis method that copes with multiple types of data as well as complex inter-nutrient relationships without subjective selection of confounders [[30], [31], [32], [33]]. Therefore, the aim of this study is to discover the interactive network between nutrients and epigenetic age acceleration by the use of CGM.

## 2 Results

### 2.1 Study sample and characteristics

Overall, nutritional and DNAm data were available for *n* = 4506 postmenopausal women. In total, *n* = 3969 participants were included in this study after the exclusion of women with more than 10% missing information in their data (*n* = 47), implausible values regarding nutrients and general covariates (*n* = 44) and duplicates (*n* = 306) (Flowchart in Supplementary files 1). In Table 1, the baseline characteristics of the study population are presented per quartile of PhenoAge acceleration

**Table 1:** The baseline characteristics of the study population stratified per quartile of PhenoAge acceleration. The nutrition, lifestyle, demographic and blood biomarker data are presented as mean (standard deviation) or percentage.

Biological Age Acceleration	Total	Q1 −33 —−4 years	Q2 −4 —0 years	Q3 0 —4 years	Q4 4 —34 years
N	3,969	995	990	992	992
Demographics (Mean [SD] or n [&])					
Age (years)	63.3 (7.1)	63.4 (7.1)	63.4 (7.2)	63.1 (7.1)	63.2 (7.0)
PhenoAge acceleration (years)	0.2 (6.4)	−7.8 (3.3)	−1.9 (1.2)	2.0 (1.2)	8.4 (3.6)
BMI (kg/m^2^)	29.6 (6.0)	28.8 (5.6)	29.1 (5.9)	30.1 (6.2)	30.5 (6.3)
Ethnicity					
* American Indian or Alaskan Native*	49 (1.2)	10 (1.0)	9 (0.9)	16 (1.6)	14 (1.4)
* Asian or Pacific Islander*	125 (3.1)	25 (2.5)	46 (4.6)	34 (3.4)	20 (2.0)
* Black or African American*	1115 (28.1)	282 (28.3)	258 (26.1)	280 (28.2)	295 (29.7)
* Hispanic/Latino*	655 (16.5)	161 (16.2)	150 (15.2)	154 (15.5)	190 (19.2)
* White*	1992 (50.2)	511 (51.4)	519 (52.4)	497 (50.1)	465 (46.9)
* Other*	33 (0.8)	6 (0.6)	8 (0.8)	11 (1.1)	8 (0.8)
Education					
*No school*	4 (0.1)	1 (0.1)	1 (0.1)	1 (0.1)	1 (0.1)
*Primary school*	162 (4.1)	44 (4.4)	38 (3.8)	42 (4.3)	38 (3.8)
*High school*	968 (24.5)	267 (27.0)	242 (24.6)	236 (24.0)	223 (22.7)
*> = Training school*	1554 (39.4)	373 (37.6)	390 (39.6)	409 (41.5)	382 (38.8)
*> = College graduate or BSc Degree*	702 (17.8)	165 (16.7)	182 (18.4)	179 (18.2)	176 (17.8)
*> = MSc Degree*	555 (14.1)	141 (14.2)	133 (13.5)	117 (11.9)	164 (16.6)
Systolic blood pressure (mmHg)	130 (18.0)	130 (18.1)	130 (18.0)	130 (18.5)	131 (17.3)
Diastolic blood pressure (mmHg)	76 (9.2)	77 (9.3)	76 (9.1)	76 (9.4)	76 (9.0)
WHR (cm)	0.8 (0.1)	0.8 (0.1)	0.8 (0.1)	0.8 (0.1)	0.8 (0.1)
Nutrients (Mean [SD])					
Caloric intake (kcal/day)	1,683 (691.7)	1,663 (698.9)	1,672 (671.9)	1,662 (665.3)	1,734 (727.2)
Carbohydrate (g/day)	201.0 (81.2)	198.6 (82.1)	199.8 (79.0)	198.7 (78.2)	207.0 (85.2)
Total sugars (g/day)	96.2 (45.9)	95.2 (43.0)	96.2 (47.7)	95.8 (46.8)	97.5 (46.1)
Protein (g/day)	68.8 (30.2)	67.2 (27.2)	68.9 (32.2)	69.5 (30.5)	69.4 (30.9)
Fat (g/day)	66.9 (35.4)	65.1 (32.6)	67.6 (38.4)	67.8 (34.7)	67.1 (35.6)
Total SFA (g/day)	22.2 (12.5)	21.5 (11.3)	22.4 (13.7)	22.6 (12.2)	22.2 (12.6)
Total MFA (g/day)	25.6 (13.9)	24.9 (12.9)	26.0 (15.1)	25.9 (13.7)	25.8 (14.0)
Total PFA (g/day)	13.8 (7.7)	13.5 (7.3)	14.0 (8.1)	13.9 (7.7)	13.8 (7.6)
Fibre (g/day)	15.1 (6.7)	15.3 (6.5)	15.3 (6.9)	15.0 (6.8)	14.9 (6.6)
Cholesterol (mg/day)	240.3 (139.7)	236.7 (139.6)	237.7 (133.5)	236.1 (136.0)	250.8 (149.0)
Vitamin A (mcg RAE/day)	741.6 (423.0)	734.3 (413.8)	728.9 (381.6)	737.9 (389.5)	765.3 (496.9)
Vitamin B6 (mg/day)	1.5 (0.7)	1.5 (0.6)	1.5 (0.7)	1.5 (0.7)	1.6 (0.7)
Vitamin B12 (mcg/day)	6.3 (4.1)	6.1 (4.0)	6.2 (4.0)	6.4 (3.7)	6.5 (4.4)
Folate Equivalents^a^ (DFE/day)	468.8 (203.1)	470.3 (194.7)	469.6 (202.3)	468.3 (212.2)	466.9 (203.0)
Vitamin C (mg/day)	93.0 (53.8)	94.7 (54.1)	93.7 (55.7)	91.0 (53.0)	92.6 (52.5)
Vitamin D (mcg/day)	4.1 (2.9)	4.0 (2.7)	4.1 (2.9)	4.2 (2.8)	4.3 (3.1)
Vitamin E (mg/day)	8.2 (5.2)	8.2 (5.2)	8.2 (5.3)	8.1 (5.3)	8.2 (5.0)
Calcium (mg/day)	766.2 (429.8)	746.1 (394.2)	771.0 (441.4)	771.8 (427.2)	775.9 (454.3)
Magnesium (mg/day)	243.5 (95.5)	243.2 (92.2)	244.5 (97.1)	243.5 (96.7)	242.6 (96.1)
Alcohol (g/day)	3.5 (8.0)	3.5 (7.8)	3.3 (7.4)	3.2 (7.8)	4.1 (8.8)
Water (g/day)	1,518 (664.0)	1,510 (655.4)	1,511 (686.6)	1,515 (645.9)	1,534 (668.1)
Caffeine (mg/day)	161.2 (139.1)	158.5 (141.2)	158.7 (135.6)	156.4 (131.9)	171.3 (147.0)
Lifestyle & disease status (N [%])					
Physical activity^b^					
* No activity*	787 (21.3)	196 (21.2)	171 (18.6)	211 (23.1)	209 (22.4)
* Some activity of limited duration*	1685 (45.6)	414 (44.8)	432 (46.9)	413 (45.2)	426 (45.6)
* 2−4 episodes p/w*	550 (14.9)	137 (14.8)	138 (15.0)	124 (13.6)	151 (16.1)
* More than 4 episodes p/w*	671 (18.2)	177 (19.2)	180 (19.5)	165 (18.1)	149 (15.9)
Smoking status					
* Never smoked*	2081 (53.2)	544 (55.2)	508 (52.2)	523 (53.6)	506 (51.7)
* Past smoker*	1469 (37.5)	368 (37.3)	367 (37.7)	363 (37.2)	371 (37.9)
* Current smoker*	363 (9.3)	74 (7.5)	98 (10.1)	90 (9.2)	101 (10.3)
Alcohol status					
* Non-drinker*	570 (14.5)	145 (14.7)	144 (14.7)	142 (14.4)	139 (14.1)
* Past drinker*	872 (22.1)	214 (21.6)	206 (21.0)	222 (22.6)	230 (23.3)
* Current drinker*	2496 (63.5)	630 (63.6)	629 (64.2)	620 (63.0)	617 (62.6)
Osteoporosis	234 (5.9)	58 (5.8)	55 (5.6)	58 (5.8)	63 (6.4)
CVD	529 (14.6)	143 (15.7)	107 (12.0)	125 (14.0)	154 (16.5)
Arthritis	1911 (48.8)	461 (46.9)	462 (47.2)	482 (49.4)	506 (51.5)
Cancer	178 (4.5)	47 (4.8)	41 (4.2)	49 (5.0)	41 (4.2)
Blood biomarkers (Mean [SD])					
N	2,185	504	579	555	547
Glucose (Mg/dl)	103.0 (31.5)	99.1 (28.1)	102.0 (28.2)	103.6 (33.4)	106.9 (35.2)
HDL Cholesterol (Mg/dl)	57.8 (15.0)	59.6 (14.8)	59.1 (15.8)	56.7 (14.4)	56.0 (14.8)
LDL Cholesterol (Mg/dl)	136.1 (35.8)	139.6 (35.5)	135.8 (36.9)	135.1 (34.9)	134.0 (35.9)
Insulin (uIU/mL)	12.3 (8.8)	10.4 (5.4)	11.6 (6.9)	12.6 (9.3)	14.4 (11.8)
Total Cholesterol (Mg/dl)	224.0 (39.4)	227.8 (40.9)	225.6 (40.6)	222.1 (36.8)	220.9 (38.9)
Triglyceride (Mg/dl)	151.5 (85.9)	143.2 (77.9)	154.5 (99.3)	154.0 (80.7)	154.0 (83.4)

Abbreviations: BMI, body mass index; WHR, waist to hip ratio; SFA, saturated fatty acid; MFA, monounsaturated fatty acid; PFA, polyunsaturated fatty acid; CVD, cardiovascular disease.

a This variable takes folic acid and natural folate into account but is largely overestimated for women who completed the WHI-FFQ prior to fortification of foods in 1997–8.

b Measure of episodes per week of moderate and strenuous recreational physical activity of > = 20 min duration (includes walking, moderate physical activity and strenuous physical activity).

(Q1: -33, -1 years; Q2: -1, 0 years; Q3: 0, 4 years; Q4: 4, 34 years).

The average chronological age of the study population was 63.3 ± 7.1 years. Most of the women were white (50.2%), overweight (BMI average of 29.6 ± 6.0 kg/m^2^), current drinkers (63.5%) and had never smoked (53.2%). Overall, almost half of the study population had a diagnosis of arthritis (48.8%). In general, the average intakes of nutrients were close to the Recommended Daily Allowances (RDAs) for older women from the U.S. stated by the Institute of Medicine. However, the average caloric intake of 1,683 kcal/day was lower than the recommended intake of around 2,000 kcal/day for women [34]. Besides, mean intakes of fibre [15.1 g/day, RDA 25−30 g/day] [35], vitamin D [4.1 mcg/day, RDA 15 mcg/day] [36], vitamin E [8.2 mg/day, RDA 15 mg/day] [37], calcium [766.2 mg/day, RDA 1,200 mg/day] [36], and magnesium [243.5 mg/day, RDA 320 mg/day] [38] were considerably lower than the RDA. By contrast, the mean intake of vitamin B12 [6.3 mcg/day, RDA 2.4 mcg/day] [39] was considerably higher than the RDA. However, the intake of nutrients via dietary supplements was not taken into account.

### 2.2 Nutritional CGM network

The main correlation matrix of the final CGM network is displayed in Supplementary File 2, where the direction of the partial correlation is multiplied by its certainty, to obtain easily interpretable values. The most important ones, i.e. the variables that are partially correlated with PhenoAge with high certainty, are summarised in Table 2. Note that all of the values presented in the figure and the table are outcomes of the CGM network and thus corrected for all other variables in the dataset. The final CGM network, including nutrients, lifestyle factors, demographics and PhenoAge acceleration, is presented in Supplementary File 2. Supplementary File 3 presents the full dataset with all certainty * partial correlation values of all pairs in the dataset. Note that only variables that partially correlate with PhenoAge acceleration in the final CGM network with a certainty of 95% or higher are described in the upcoming sections and in Table 2. A positive correlation indicates that higher intake of the nutrient relates to PhenoAge acceleration (i.e., increased ageing rate), whereas a negative correlation relates to PhenoAge deceleration (i.e., decreased ageing rate).

**Table 2:** Overview of variables with an over 95% certain direct correlation with PhenoAge acceleration in the main CGM network, and an overview of their robustness over several sensitivity analyses.

AMIN, American Indian or Alaskan Native; ASIA, Asian or Pacific Islander; Cer, certainty; Cor, direction of correlation; Hisp, Hispanic or Latino.

^1^Ash intake represents the intake of inorganic mineral content, and is mainly determined by dietary sodium.

Intakes of coumestrol, beta-carotene and SFA 20:0 (arachidic acid) were directly related to PhenoAge deceleration. Intakes of added sugar, MFA 20:1 (gondoic acid), SFA 22:0 (behenic acid) and PFA 20:4 (arachidonic acid), vitamin A and ash (inorganic mineral content, mainly sodium) were directly related to PhenoAge acceleration. These nutrient-ageing relationships were confirmed in the several sensitivity analyses that were performed. The certainties of these strata-specific estimates were always ≥80% and showed the same directions as in the main network, except for ash intake, where the finding was not replicated in more than half of the sensitivity analyses.

Smoking, BMI, WHR, and Systolic blood pressure were related to PhenoAge acceleration and were robust over the different sensitivity analyses. However, in the participants without arthritis, the smoking – PhenoAge relationship was in an opposite direction. Asian, American Indigenous, Hispanic and Other were related to PhenoAge acceleration but were not robust in the various sensitivity analyses. Higher diastolic blood pressure was consistently related to PhenoAge deceleration. The presence of osteoporosis, cancer, and cardiovascular diseases were related to decelerated ageing in all networks except in the younger half of the cohort for all three diseases, and in the lower BMI half for cardiovascular diseases. Presence of arthritis related to PhenoAge acceleration in the main network but not in four out of seven sensitivity analyses.

### 2.3 Blood biomarkers

In around half of the participants, blood biomarkers were measured. These biomarkers were tested in a separate network analysis, including all other variables. Blood levels of LDL cholesterol and glucose were related to PhenoAge acceleration, with 100% and 96% certainty, respectively. Blood levels of triglycerides and HDL cholesterol were related to PhenoAge deceleration, with 99% and 95% certainty, respectively.

## 3 Discussion

This study aimed to identify nutrients that relate to decelerated biological ageing in a preregistered, data-driven way, while rigorously correcting for every other variable in the dataset. Several direct relations were revealed. Dietary intakes of coumestrol, beta-carotene and arachidic acid were discovered to be negatively associated with PhenoAge acceleration, thus leading to a favourable lower ageing rate. On the other hand, intakes of gondoic acid, arachidonic acid, behenic acid, and added sugar were discovered to be positively associated with PhenoAge acceleration. Apart from nutrients, clear ageing-acceleration associations were found for smoking, BMI, WHR and systolic blood pressure, and an ageing-deceleration relationship was found for diastolic blood pressure.

A striking finding was the negative association between PhenoAge acceleration and dietary coumestrol intake, a phytoestrogen extracted from soybeans [40]. Dietary intake of coumestrol in our participants mainly originated from the intake of dairy products, soy milk, vegetables and various types of meat. Given the increasing scientific interest in the anti-ageing effects of phytoestrogens, coumestrol appears to have promising therapeutic applications as an antioxidant, although the underlying molecular mechanism remains unclear [40]. Moreover, several in vitro and rodent studies showed possible protective effects against postmenopausal disorders, such as CVD and diabetes, due to the estrogen-mimicking effects of coumestrol treatment [41,42]. These estrogen-mimicking effects may explain coumestrol’s association with decelerated biological ageing in our sample of postmenopausal women, but we should cautiously interpret these findings in the light of breast-cancer risk increasing results of estrogen and progestin treatment in the WHI intervention trial [43]. It would be interesting to repeat our analyses in samples of men or premenopausal women to see if coumestrol intake relates to ageing independent of menopausal status.

Our study revealed that beta-carotene intake associated with PhenoAge deceleration, a beneficial finding that aligns with previous studies. Blood levels of carotenoids have been associated with decelerated biological ageing before [10,44,45], and growing evidence suggests that beta-carotene acts as an anti-ageing agent through its antioxidant mechanism [46]. A meta-analysis found an inverse association between beta-carotene and age-related disease, suggesting that beta-carotene could be a beneficial nutrient in healthy ageing [47]. Our results add confidence to the idea that a diet rich in beta-carotene-containing products, such as fruits and vegetables, may be associated with slower epigenetic ageing.

The discovery that vitamin A intake is associated with accelerated PhenoAge seems conflicting, particularly since beta-carotene—a precursor of vitamin A—serves a similar physiological role in the body. Interestingly, these two nutrients are sourced from distinctly different foods: beta-carotene mainly from plants, and vitamin A exclusively from animal-based foods such as organ meats. Previous studies have associated organ meat and red meat intake with non-alcoholic fatty liver disease [48] and various age-related diseases [49], respectively. Moreover, both beta-carotene and vitamin A supplementation have been linked to increased lung cancer risk, although this seems limited to heavy smokers [50]. Our analysis reveals a nuanced picture. While one might expect beta-carotene and vitamin A to have similar effects on ageing due to their physiological similarities, the data suggest otherwise. This divergence can be explained more by their dietary sources than by their biological functions. It serves as a reminder that observed associations between specific nutrients and ageing do not necessarily imply a direct biological mechanism affecting age-related processes. Instead, these nutrients might be markers for broader dietary patterns or even for the intake of yet-unmeasured compounds like certain flavanols or sialic acids. Importantly, these associations are adjusted for all available dietary and lifestyle variables, ruling out the possibility that beta-carotene and vitamin A are simply proxies for other elements like iron, saturated fatty acids, or fibre in our diet.

Interestingly, differing associations per fatty acid with PhenoAge acceleration were discovered, while no (certain) association was found with the total intake of saturated fatty acids (SFA), monounsaturated fatty acids (MFA), or polyunsaturated fatty acids (PFA). Commonly, fatty acids are studied in broad subgroups. As indicated by the results of this and other studies, it seems prudent to use a more detailed classification to study fatty acid intake and health. In this study, arachidic acid, a member of very long chain saturated fatty acids (VLSFAs) and a component of peanut oil, was the only fatty acid associated with PhenoAge deceleration. While associations between blood VLSFA concentrations and reduced risk of CVD have been reported [51], we are unaware of any study that investigated specifically arachidic acid related to ageing or age-related diseases. Moreover, behenic acid, another VLSFA and a component of several oils, was found to be positively associated with PhenoAge acceleration in our study, which is in line with one clinical study that found that behenic acid raises total and LDL cholesterol [52], indicating that grouping fatty acids on saturation and chain length might not be detailed enough.

Gondoic acid, a constituent in several plant oils [53], and arachidonic acid, an omega-6 fatty acid found in animal products [54], were also related to accelerated biological ageing. To our knowledge, only one observational study found a positive association between blood gondoic acid concentrations and all-cause and cardiovascular mortality risk in women, which may support our finding [53]. Lastly, in the case of arachidonic acid, previous research is yet indecisive about the link between excessive inflammation, which accelerates ageing, and high omega-6 in the diet [[55], [56], [57]]. Yet, in accordance with the inconclusive evidence, our findings underline the importance of investigating fatty acids as separate nutrients instead of as a group.

Overconsumption of sugar is related to various adverse health effects [58,59]. However, it is crucial to differentiate sugar types as the consumption of whole foods that naturally contain sugar is essential for overall health. Our study showed that 'added sugar', and not total sugar or total carbohydrate intake, was associated with PhenoAge acceleration. This aligns with an intervention study that linked sugar-sweetened beverages, the primary source of added sugar, to accelerated ageing and increased risks of cardiometabolic diseases [60]. Intake of added sugar has been associated with progressive telomere shortening via oxidative stress and inflammation [58,60]. Therefore, our research finding supports the idea that added sugar accelerates ageing and emphasises the importance of limiting intake. Similarly, ash intake, which represents inorganic mineral content and thus largely sodium content of foods, was expectedly related to accelerated biological ageing, albeit without robustness over the sensitivity analyses. Conversely, alcohol intake did not demonstrate a significant relationship with biological aging in our study, which contradicts the findings reported in the Framingham Heart Study [61]. This discrepancy may be attributed to cohort-specific factors, as our cohort consists of postmenopausal women who may have altered their alcohol consumption patterns over time. Our analysis considered only current alcohol intake measured in grams, potentially overlooking the impact of past alcohol consumption. This is particularly relevant for individuals who were former heavy drinkers and have since ceased drinking, as their historical alcohol intake may have exerted a more substantial influence on age acceleration.

This study looked at nutrient intake obtained from FFQs, which should reflect the usual intake over the previous months. Dietary assessment is typically prone to underreporting, and the low average caloric intake reported by our participants suggests that underreporting was indeed present in our study. The FFQ used in WHI showed to be valid against a four-day food record but has not been validated against objective measures such as energy expenditure measured by doubly labelled water [62]. Selective underreporting of unhealthy foods may have led to an underestimation or misidentification of nutrients. Importantly, nutrient intakes were calculated from foods only and were never derived from supplement intakes. The availability of a broad range of nutrients and food components, including individual fatty acids, enables us to sharply pinpoint the source of associations. However, still many of the bioactive components in foods remained unavailable, raising the possibility that compounds may have been missed or that nutrients highly correlated to such unquantified compounds were erroneously identified.

The identification of known age-accelerating factors, smoking, BMI, WHR, systolic blood pressure, blood LDL-cholesterol concentrations and blood glucose concentrations, increases the confidence in the validity of the final network. Also, the stability of the findings over several sensitivity analyses underlines the robustness of the findings. Striking results, such as the relationship between coumestrol intake and decelerated biological ageing, were observed consistently across the separate networks of the two study cohorts used in the analyses. It would be interesting to assess their reproducibility in a separate cohort in which similar nutrients are measured.

The role of ethnicity in biological ageing remains complex and multifaceted. Ethnic differences are known to influence epigenetics [63], potentially affecting dietary habits and reflecting broader, unmeasured socio-environmental factors. In our analysis, certain ethnicities appeared to correlate with accelerated biological ageing. However, this association demonstrated limited robustness and was predominantly observed in individuals with a higher BMI and those suffering from arthritis. Additionally, our data clearly illustrated the significant impact of age-related diseases such as osteoporosis, cancer, and cardiovascular disease on DNA methylation. These diseases disrupt epigenetic processes, including those related to DNA methylation clocks, which could contribute to altered ageing trajectories [64,65], explaining the unexpected link observed between these diseases and a seeming deceleration in the ageing process.

This study benefited from preregistration at OSF, which increased the credibility and transparency of the results. The preregistered protocol was exactly followed except for (1) the additional cohort-based sensitivity analyses and (2) the argument setting in CGM of *rho,* which we changed into a range rather than a fixed number in order to select the optimal penalty term. The use of an epigenetic clock to estimate biological age is a main strength of this study, and in particular PhenoAge, which is based on a specific set of CpG-sites related to multifactorial phenotypic ageing [25].

By using CGM, we not only confirmed previously identified relationships between PhenoAge acceleration and various nutrients, lifestyle factors, and demographics but also discovered new nutrients correlated with epigenetic ageing rates. Our findings reaffirm CGM’s utility as a powerful identification tool in nutritional research. Unlike traditional methods, such as linear regressions, which require pre-selection of exposures, outcomes and confounders, CGM can handle multiple types of data commonly found in nutritional datasets, such as and non-Gaussian, mixed discrete-continuous data, and adjusts for all variables in the dataset using conditional dependency [30,31,33]. Moreover, although our approach may appear reductionistic, it does account for the relationship of a single nutrient while considering all other nutrients in the database, thereby highlighting the significance of overall dietary patterns.

However, while CGM successfully identifies direct relationships, it provides no usable information on the clinical relevance of these relationships [30,33]. For instance, to find out how much coumestrol intake is needed to obtain meaningful differences in PhenoAge deceleration, multiple linear regression should be performed with the inclusion of the covariates that our research also identified. Moreover, despite CGM's adjustment of correlations between two variables for all other variables in the dataset, residual confounding remains possible via unmeasured or excluded variables. We tried to alleviate this limitation by including 139 variables, but still, we would have benefitted from more variables, for instance, on other bioactive compounds in foods.

In conclusion, we discovered several direct relations between epigenetic ageing and nutrients using the powerful objective statistical method of CGM. We revealed that dietary intakes of coumestrol, beta-carotene and arachidic acid were associated with decelerated epigenetic ageing, whereas intakes of added sugar, gondoic acid, behenic acid, arachidonic acid, vitamin A, and ash were associated with accelerated epigenetic ageing in postmenopausal women. Our study provides novel hypotheses on nutritional factors potentially accelerate or decelerate epigenetic ageing, which should be tested in other populations and cohorts and via various research designs.

## 4 Methods

### 4.1 Participants and study design

The current study presents a secondary analysis of the Women's Health Initiative (WHI). The WHI is a large long-term health project in the U.S. that investigates strategies for the control and prevention of causes of morbidity (e.g., cancer, CVD, osteoporotic fractures) and mortality among postmenopausal women [66]. This national study started in 1992 and enrolled postmenopausal women into either a clinical trial or an observational study (OS) within the WHI. Based on an eligibility screening questionnaire, study participants were excluded if they had medical conditions predictive of a survival time of less than three years; if they were known to have conditions or characteristics inconsistent with study participation and adherence (alcoholism, drug dependency, mental illness, dementia); or if they were active participants in another clinical trial [66]. A detailed description of relevant details including the design of WHI and the participant recruitment has been documented by the WHI study group [66].

This study aggregated cross-sectional data from participants in two WHI ancillary studies (BA23 and AS315) to discover specific nutrients that are linked to epigenetic ageing. We examined all the ancillary study participants with available baseline DNAm data, dietary information, lifestyle habits and demographic data. In aggregate, that total included 4,506 postmenopausal women of different races and ethnicities (e.g., White, African Americans, Hispanics) and a chronological age between 50 and 79 years at baseline [66]. The two WHI ancillary studies providing epigenetic data were: (1) WHI-BA23 (*Integrative Genomics and Risk of CHD and Related Phenotypes; n =* 2,108), a case-control study assessing genomic predictors of coronary heart disease (CHD) using eight biomarkers of CHD and genomic procedures [67]; and (2) WHI-AS315 (*Epigenetic Mechanisms of Particulate Matter-Mediated CVD Risk; n =* 2,399), a study investigating the epigenetic mechanisms underlying the association between ambient particulate matter (PM) air pollution and CVD via genome-wide analysis within a center- and race/ethnicity-stratified, random minority oversample of WHI clinical trial participants with core analytes [67].

In February 2023, the study was preregistered at Open Science Framework (OSF) in order to create transparency and reduce bias upon our exploratory research approach. To ensure objectivity in this study, a strict research protocol and detailed description of our study design, data use and statistical analyses was submitted prior to statistical analyses. The preregistration can be viewed using the following link (https://doi.org/10.17605/OSF.IO/J8M6P).

### 4.2 Data collection

#### 4.2.1 General covariates

Participants completed self-administered questionnaires at baseline between 1993 and 2005 which provided personal information on a wide range of topics, including sociodemographic information, personal habits and medical status. Additionally, participants visited clinics at baseline where certified Clinical Centre staff performed anthropometric measurements including weight, height, hip and waist circumferences, and systolic and diastolic blood pressures; body mass index (BMI) and waist to hip ratio (WHR) were calculated from these measurements [10].

#### 4.2.2 Dietary intake

The total dietary intake of each participant was estimated using the self-administered WHI Food Frequency Questionnaire (FFQ) [62]. At baseline between 1993 and 2005 the WHI-FFQ was mailed by the Clinical Centre staff to all participants and completed and returned by the participants. The main purpose of the WHI-FFQ was to assess dietary habits of the past three months in order to estimate intake of selected nutrients [10,62]. Note that nutrient intake from dietary supplements was not taken into account [62]. Regarding the three sections on food, section one consisted of nineteen adjustment questions about food preparation methods and types of fat added to foods, to ensure more tailored analysis of nutrient content. The main section (food items) consisted of questions on intake frequency and portion size of 122 foods or food groups. The last section consisted of four summary questions about habitual consumption of fruits, vegetables, and fats used in cooking [62]. The WHI-FFQ has a specific focus on low-fat foods, because one WHI clinical trial involved a low-fat intervention. Given the multi-ethnicity of the WHI participants, the FFQ also reported on regional and ethnic eating patterns [62]. To ensure quality of the questionnaire outcomes, 90% of the questions of the main section (food items), at least half of every subsection (e.g., fruits and vegetables) and all adjustment and summary questions should be answered. If less than 90% of the food items was completed, the reviewer declared the FFQ unacceptable and returned it to the participant for completion [62]. Validation studies in which the WHI-FFQ was compared with other dietary assessment methods, such as food records or 24 -h recalls, showed similar nutrient estimates (mean intakes as measured by the WHI-FFQ were within 10% of the record/recall estimations) [62]. Moreover, the WHI-FFQ is considered to be a similar or even better measurement of dietary intake compared to other FFQs in similar populations [62]. In 2005, after baseline measurements, the nutrient intake values were calculated from the WHI-FFQ responses by the Fred Hutchinson Cancer Center by Nutrition Assessment Shared Resource (NASR) utilising the Nutrition Data System for Research (NDSR) software developed by the Nutrition Coordinating Center (NCC). The NCC food and nutrition database provides over 140 nutrients and bioactive compounds [62].

#### 4.2.3 Blood biomarkers

Participants in both WHI ancillary studies (BA23 and AS315) from the WHI had baseline blood specimens collected by certified Clinical Centre staff after an overnight fast in ethylenediaminetetraacetic acid (EDTA) tubes and stored at −70℃. These samples were processed at the WHI core laboratory and select blood biomarkers were measured including triglyceride, total, LDL, and HDL cholesterol [10].

#### 4.2.4 DNA methylation and epigenetic clock

DNAm data were obtained from the blood samples [10]. Firstly, 500 ng of genomic DNA was extracted from each blood sample. Subsequently, the DNA samples underwent bisulfite treatment to mediate the deamination of cytosine into uracil, which was read as thymine. However, the remaining 5mC are resistant to this conversion and remain readable as cytosine. Lastly, the bisulfite-converted DNA was used for the genome-wide DNAm analysis which was done with the Infinium HumanMethylation 450 BeadChip (Illumina). This platform used bisulfite conversion to quantify methylation levels at 485,577 specific CpG-sites [10,24,68]. As cytosine can either be methylated (noted as 1) or unmethylated (noted as 0), the returned value represented the proportion of methylation of a given CpG-site in a population of blood cells as a value between 0 and 1 [69]. By using these proportions, the epigenetic clock estimates biological age from the DNAm data from the blood samples [24,68,69].

In BA23, DNAm was measured using the Illumina at the HudsonAlpha Institute of Biotechnology, and in AS315 using Illumina at Northwestern University Genomics Core Facility [10]. In order to test the quality of the BA23 array measurements, correlation measures were performed with duplicates within this dataset and with a “gold” standard which is an average of many samples previously collected. Correlation between duplicates and with the gold standard were high (r>0.9), indicative of high-quality measurements. To evaluate the reliability of the AS315 DNA methylation measurements, site-specific variance was analyzed using random-effects ANOVA and intra-class correlation coefficients (ICC) [70], with further validation through bisulfite pyrosequencing in a subset of participants [71], all confirming high-quality and consistent results. The DNAm data corresponding to the WHI participants were available following the WHI SharePoint page (BA23) and via the WHI Secure Research Workspace at the University of North Carolina (AS315).

In this study, biological age was estimated using an established epigenetic clock, PhenoAge [25]. This epigenetic biomarker of ageing is based on 513 CpG-sites from whole blood DNAm data and estimates multifactorial phenotypic age comprised of ten phenotypic ageing measures, including chronological age, glucose and creatinine level [25]. The clock was trained and validated by studies in multiple independent and large cohorts, demonstrating that PhenoAge is an accurate biomarker of ageing and a strong predictor of mortality and morbidity outcomes [25]. Moreover, in comparison to previously developed clocks (e.g., Horvath) that use chronological age as a reference, PhenoAge appeared to be better in predicting mortality, physical functioning and disease [22,25]. PhenoAge is a holistic measure due to its unique combination of phenotype measures and specific DNAm sites to estimate biological age and predict age-related disease and mortality. Therefore, it was considered as the most optimal clock for identifying nutrients that modify the ageing process. Although the clock was developed using whole blood samples, it strongly correlates with age among a wide variety of cells and tissues and is therefore able to estimate tissue-specific ageing [25]. In order to estimate biological age with PhenoAge for each participant, an online biological age calculator was used in which DNAm values of selected CpG-sites of all the subjects were uploaded https://dnamage.genetics.ucla.edu/. Subsequently, this online software tool automatically measured PhenoAge acceleration (i.e., epigenetic age acceleration) for each participant by calculating the relative discrepancy between chronological age and biological age estimated by PhenoAge. This calculation was derived by regressing PhenoAge on chronological age, and the residuals of that linear regression were used as the measure of PhenoAge acceleration.

### 4.3 Statistical analysis

#### 4.3.1 General covariates and nutrients

First, dietary information from the WHI-FFQ was considered unreliable and excluded from further analysis if reported caloric intakes were <600 kcal/day or >5,000 kcal/day. Second, in order to identify outliers in our data, boxplots per variable were generated to obtain a visual presentation of the data. Based on these boxplots, data points outside the lower and upper bound range of 1.5 times the interquartile range (IQR) were identified as outliers where values were unrealistic or implausible to be obtained from dietary intake, lifestyle or demographics. Accordingly, participants with implausible values regarding nutrients and general covariates were excluded from further analysis (n = 329). Lastly, prior to network analysis, variables as well as participants with more than 10% missing values coded as 'NA' were deleted from the dataset to improve parameter estimation, model selection, computational efficacy and reduce bias in CGM.

#### 4.3.2 Copula graphical models

To discover the direct relations between nutrients and PhenoAge acceleration, graphical models (GM) were used. GMs are a combination between graph theory and probability theory, offering a robust approach for dealing with complex data [30]. In this study, the undirected type of GMs (UGM) was used which comprises probability structures that depict the conditional (in)dependence relationships among all variables in the dataset [72]. These conditional (in)dependence relationships among random variables can be summarised in a graph with a set of nodes in which each node corresponds to a random variable [30,72]. Any pair of nodes can be joined by an undirected edge, representing symmetric relations, while the absence of an edge indicates a form of independency between a pair of nodes (variables) [30,72].

In this study, the use of UGM is preferred since this statistical tool enables a data-driven approach allowing for an objective exploratory analysis in this field of research where too little information for a hypothesis-driven approach is available [30]. The implemented UGM is able to uncover conditional dependence relationships between random variables by means of penalty inference based on the penalised Expectation-Maximization (EM) algorithm [30,33,73]. As such, UGM corrects for the effect of every other variable incorporated in the model while the conditional dependence relationship between two variables is measured [72]. That means that every pairwise association presented in the results is adjusted for all the other variables included in the dataset, without any selection or weighing.

Therefore, in comparison to other exploratory statistics, such as Pearson correlations, which depict marginal (in)dependencies, no prior selection of dependent relationships or confounders is needed [30], but rigorous confounder adjustment is implemented.

The simplest possible type of UGM are Gaussian Graphical Models (GGM) as the conditional dependence relationships can be derived from the inverse of the variance-covariance matrix and contains exclusively Gaussian distributed data [33]. Copula Graphical Models (CGM) are a flexible type of UGMs in which non-Gaussian distributed data can be applied as well [30,72]. In fact, CGM deals with multiple types of data, such as categorical and ordinal data, as well as missing data and uses the lowest possible number of parameters to describe the full multivariate dependence structure [30,32]. Besides, CGM is able to deal with multicollinearity between variables in a model. As mixed types of variables and multicollinearity between variables are common in nutrition datasets, CGM is a suitable method to explore the direct relations between PhenoAge acceleration, nutrition, lifestyle factors and demographics in our present study [30].

The statistical analysis was performed in R version 4.1.3 for Windows with the R package *nutriNetwork*, which was specifically designed for CGM [33,74]. *nutriNetwork* measures the conditional dependence relationships between all variables and depicts them as partial correlations in a graphical network [30]. The *nutriNetwork* package was designed upon a sparse network including more variables than subjects (i.e., n < p). However, in this study the data includes more subjects than variables (i.e., n > p) asking for a lower penalty term close to zero in order to create a sparse network. Accordingly, the setting of *rho* was a range between 0.01 to 0.001 in which *nutriNetwork* selected the optimal *rho*. The *nutriNetwork* analysis was run with the following settings [30,33].

*rho <- seq(0.01, 0.001, length.out = 20)*

*nutriNetwork(data, method = "npn", rho = rho, rho.ratio = NULL)*

Following arguments:

- Data: An (n × p) matrix, where n is sample size and p is the number of variables. Input data can contain missing values.
- Method: Three methods are available to generate graphical networks, namely Gibbs sampling (Gibbs), approximation method (approx.), and nonparanormal approach (npn) within CGM.
- Rho: A decreasing sequence of non-negative numbers that control the sparsity level.
- Rho.ratio: Determines the distance between the elements of rho sequence.

All available arguments are outlined by the description manual of *nutriNetwork* R package [30].

To compute the optimal network model for our dataset, the selectnet function in R was used. Selectnet uses the extended Bayesian information criteria (eBIC) to select the most optimal network model [33].

*FinalNetwork <- selectnet(nutriNetwork.obj)*

Following arguments:

- FinalNetwork: stores the function output.
- nutriNetwork.obj: the nutriNetwork output.

*NutriNetwork* generates multiple network models based on varying penalty terms resulting in different degrees of sparsity [30,33]. Selectnet picks the graph estimation with the lowest eBIC value in order to select the ultimate penalty term leading to an optimal degree of sparsity [30,33].

#### 4.3.3 Certainty analysis

To determine the underlying statistical (un)certainty and importance of each partial correlation in our estimated diet-epigenetic network, a bootstrap method was used [33]. This approach created resampled datasets which were generated by sampling of replacements using the values of our initial dataset [33]. By doing so, multiple bootstrap samples were created and the entire CGM procedure including the optimal network selection was applied to each newly obtained dataset [30]. We resampled our data a total of 100 times. The frequency of each identified partial correlation across these resampled datasets presented the underlying certainty in the diet-epigenetic network and the statistical significance that this correlation exists in this population [30,33]. The partial correlations with PhenoAge acceleration that presented a certainty of 95% or higher were eventually included in this study.

#### 4.3.4 Sensitivity analysis

To determine the sensitivity of correlations within the estimated network, separate CGM networks were created on data split on three baseline characteristics: BMI, chronological age and arthritis. Per characteristic the data was separated on the median of BMI 28.8 kg/m^2^ (BMI =< median vs BMI > median) and on the median of chronological age 63 years (age =< median vs. age >median), and on presence of arthritis (no arthritis vs arthritis). These characteristics may have affected diet-epigenetic direct relations to some extent based on previous research and our baseline table (Table 1) [25]. To test observed partial correlations in the final CGM network, the same network stratified per subgroup of characteristic was generated. Additionally, as blood biomarkers were only available for ∼55% of the study population, a separate CGM diet-epigenetic network was generated based on participants with available blood biomarker data.

## Funding

The Women's Health Initiative (WHI) programme is funded by the National Heart, Lung, and Blood Institute, National Institutes of Health, US Department of Health and Human Services through contracts 75N92021D00001, 75N92021D00002, 75N92021D00003, 75N92021D00004, 75N92021D00005. WHI investigators are listed @ https://www.whi.org/doc/WHI-Investigator-Long-List.pdf. WHI data are publicly available @ https://www.whi.org/md/working-with-whi-data. AS315 was funded by National Institute of Environmental Health Sciences grant R01-ES020836. BA23 was funded by the National Heart, Lung, and Blood Institute Broad Agency Announcement contract HHSN268201300006C.

P.G. was supported by a grant 162135 from the Regio Deal Foodvalley; S.H. was supported by grant 1U01AG060908-01 from National Institute on Ageing–National Institutes of Health; C.S.K. was supported by grant 457001001 from ZonMW; J.E.M was supported by HL34594, HL145386 and 75N92021D00002 from the National Heart, Lung, and Blood Institute; A.H.S. was supported by grant RF1AG074345 from the National Institute on Aging; J.D.S. was supported by grant R01ES020836 from the National Institute of Environmental Health Sciences and contract 75N92019R0031 from the National Heart, Lung, and Blood Institute; E.A.W. was supported by grant R01ES020836 from the National Institute of Environmental Health Sciences and contract 75N92019R0031 from 10.13039/100000050the National Heart, Lung, and Blood Institute.
