# Supplementary material for: Discovering the direct relations between nutrients and epigenetic ageing
Source: J Nutr Health Aging. 2024 Jul 26;28(9):100324. doi: 10.1016/j.jnha.2024.100324 (PMC12880071; doi:10.1016/j.jnha.2024.100324)
Supplement: Supplementary file 1 [file mmc1.docx]

# **Supplementary files**

## *Supplementary file 1 | Flowchart of participants*

**Women’s Health Initiative (WHI)**

(n=378 571)

AS315 with cleaned nutritional and DNAm data

(n=1957)

**Excluded** (n=442)

- Incomplete DNAm data (n=7)
- Duplicates (n=200)
- Implausible nutritional data (n=235)

**Excluded** (n=95)

- Incomplete DNAm data (n=1)
- Implausible nutrition data (n=94)

BA23 with cleaned nutritional and DNAm data

(n=2012)

**Excluded** (n=221 660)

- Caloric intake <600/day or >5000/day (n=11,759)
- Measurement not performed at T=0 (n=209,901)

**Excluded** (n=152 405)

- No combination of DNAm data and nutrition data

BA23

(n=2107)

AS315

(n=2399)

Analytical study population

n = **3969**

WHI

(n=156 911)

## *Supplementary file 2 | The correlation*certainty matrix*

This correlation matrix shows the direction of the correlation (green= negative, red= positive, open= uncorrelated) and the certainty of the correlation, where 50% is a half-circle and 100% is a full circle. The correlations shown depict the partial correlation values between all the nutrients, demographics, lifestyle factors and PhenoAge acceleration from the final CGM network. It can be accessed using the following link:

[https://doi.org/10.6084/m9.figshare.23696607.v2](https://doi.org/10.6084/m9.figshare.23696607.v2%20)

## *Supplementary file 3 | The dataset*

The dataset that includes the values for [direction of correlation (-1, 0 or 1)] * [certainty of correlation (0-100% based on bootstrapping)]. The actual value of the partial correlation holds negligible meaning in a Copula Graphical Model, and therefore, it has been reduced to its direction. The direction, in combination with the certainty of identifying the partial correlation between two variables over 100 iterations of the network, is more meaningful and shown in this dataset. It can be accessed using the following link.

<https://figshare.com/s/d450e71eec16eb81f8eb>
